# Expression Profile of *PIN*-Formed Auxin Efflux Carrier Genes during IBA-Induced In Vitro Adventitious Rooting in *Olea europaea* L.

**DOI:** 10.3390/plants9020185

**Published:** 2020-02-03

**Authors:** Isabel Velada, Hélia Cardoso, Sara Porfirio, Augusto Peixe

**Affiliations:** 1 MED—Mediterranean Institute for Agriculture, Environment and Development, Instituto de Investigação e Formação Avançada, Universidade de Évora, Pólo da Mitra, Ap. 94, 7006-554 Évora, Portugal; 2 Complex Carbohydrate Research Center, The University of Georgia, 315 Riverbend Road, Athens, GA 30602, USA; 3 MED—Mediterranean Institute for Agriculture, Environment and Development & Departamento de Fitotecnia, Escola de Ciências e Tecnologia, Universidade de Évora, Pólo da Mitra, Ap. 94, 7006-554 Évora, Portugal

**Keywords:** olive, auxin, plant propagation, PIN, adventitious roots, reactive oxygen species (ROS), wounding, gene expression

## Abstract

Exogenous auxins supplementation plays a central role in the formation of adventitious roots (AR) for several plant species. However, the molecular mechanisms underlying the process of adventitious rooting are still not completely understood and many plants with economic value, including several olive cultivars, exhibit a recalcitrant behavior towards cutting propagation, which limits its availability in plant nurseries. PIN-formed proteins are auxin efflux transporters that have been widely characterized in several plant species due to their involvement in many developmental processes including root formation. The present study profiled the expression of the *OePIN1a-c*, *OePIN2b*, *OePIN3a-c*, *OePIN5a-c*, *OePIN6*, and *OePIN8* gene members during indole-3-butyric acid (IBA)-induced in vitro adventitious rooting using the olive cultivar ‘Galega vulgar’. Gene expression analysis by quantitative real time PCR (RT-qPCR) showed drastic downregulation of most transcripts, just a few hours after explant inoculation, in both nontreated and IBA-treated microcuttings, albeit gene downregulation was less pronounced in IBA-treated stems. In contrast, *OePIN2b* showed a distinct expression pattern being upregulated in both conditions, and *OePIN5b* was highly upregulated in IBA-induced stems. All transcripts, except *OePIN8*, showed different expression profiles between nontreated and IBA-treated explants throughout the rooting experiment. Additionally, high levels of reactive oxygen species (ROS) were observed soon after explant preparation, decreasing a few hours after inoculation. Altogether, the results suggest that wounding-related ROS production, associated with explant preparation for rooting, may have an impact on auxin transport and distribution via changes in *OePIN* gene expression. Moreover, the application of exogenous auxin may modulate auxin homeostasis through regulation of those genes, leading to auxin redistribution throughout the stem-base tissue, which may ultimately play an important role in AR formation.

## 1. Introduction

Auxins are an important group of phytohormones that influence cell division, cell elongation, and cell differentiation and thus have been widely reported to be involved in the control of plant growth and development [1,2,3]. Several studies have been performed on the involvement of auxins, for example, in the regulation of zygotic embryo development, [4], general shoot and root development and architecture [5,6,7,8,9], tropisms [10,11,12], temporal coordination of plant tolerance to stress [13,14,15], apical dominancy regulation [16,17], development of leaves and flowers [18,19], nodulation [20], or organ initiation at the shoot apex [21,22,23].

Although auxins were long believed to be mainly synthesized in the young leaves and apical meristem of the shoot [24], more recent studies found that both shoots and roots can produce auxins [25]. From the primary biosynthesis sites, auxins are distributed throughout the plant by two interconnected transport systems, one consisting of a fast nondirectional stream in the phloem along with photosynthetic assimilates, and the other comprising the polar auxin transport (PAT) system [1]. PAT is the major auxin distribution pathway, dispersing auxin from cell-to-cell in a slow and direct manner, and thus is determinant for the spatio-temporal dissemination of that molecule within tissues during plant development. This is achieved by establishing defined auxin gradients (auxin homeostasis), involving local auxin maxima or local auxin optima, which inhibit and promote, respectively, cell division, expansion, or differentiation [1,26,27,28,29]. There are three main classes of auxin membrane transporters mediating the PAT system: the influx carriers auxin permease 1 (AUX1)/LAX, and two classes of efflux carriers, namely (1) the ATP-BINDING cassette subfamily B (ABCB; previously known as multidrug resistance (MDR)/Phosphoglycoprotein (PGP)) transporters, and (2) the PIN-formed (PIN) proteins [27].

In Arabidopsis, the PIN family consists of eight members grouped into two subclasses according to the length of their central hydrophilic loop. Canonical ‘long’ or ‘PIN1-type’ proteins (PIN1, PIN2, PIN3, PIN4, and PIN7) localize to the plasma membrane (PM). Nevertheless, they do not reside statically in the PM but constitutively cycle between the PM and endosomal compartments. The three remaining members of the AtPIN family display a partly (PIN6) or dramatically reduced (PIN5 and PIN8) central hydrophilic loop (reviewed by [30]). The PIN efflux carriers are being widely characterized in several plant species and various functions have been attributed to PIN proteins in plants. Among these are functions related to root initiation and development, as is the case of PIN1 [31], PIN3, PIN5, and PIN6 (reviewed by [32]).

The formation of adventitious roots (AR) is a developmental process where new roots arise from stems, leaves, or non-pericycle tissues in old roots. AR may be formed naturally or under stressful environmental situations, and they may be also induced by mechanical injury or following tissue culture regeneration of shoots [33,34,35]. The process of AR formation has been studied in olive (*Olea europaea* subsp. *europaea* var. *europaea* L.) (reviewed by [36]) because this crop comprises several cultivars with reduced capacity to be propagated given its recalcitrance towards adventitious rooting. The cultivar ‘Galega vulgar’ is one of those cultivars, displaying a difficult-to-root behavior. Microcuttings of this cultivar treated with IBA (indole 3-butyric acid) have been used to study the molecular mechanisms underlying the formation of AR. The olive adventitious rooting process is considered a developmental process organized in a sequence of interdependent stages, including (1) an induction phase corresponding to the first 4 days after microcutting treatment and inoculation; (2) an initiation phase, from 4 to 14 days after treatment, when the first meristemoids and morphogenetic root zones are observed; and (3) the expression phase, which starts at 22 days after the root-inducing treatment [37]. 

Auxins play a central role in the control of adventitious rooting [38,39,40,41], and for that reason are widely used as an inducer of the process. Indeed, auxin transport, particularly through PIN auxin carriers, is a key factor in AR formation [31,42,43]. AR in olive cuttings has been suggested as a direct growth response, involving a stress-induced reprogramming of shoot cell fate by which plants diminish stress exposure [44,45,46]. Another group of signaling molecules described as central players in the coordination of responses to biotic and abiotic stresses in plants, as well as in the regulation of plant growth and development, are reactive oxygen species (ROS) [47]. The existence of cross-talk between ROS and auxin homeostasis and signaling has been largely reported [48,49,50,51,52,53,54,55,56], under the assumption that, for example, ROS can alter directional transport of auxin by inducing auxin-conjugating enzymes, or reducing the transcription of auxin-responsive genes.

Despite the great attention that has been given to the PIN-formed auxin efflux transporter genes in many plant species, both in model and crop plants (reviewed by [32]) and in many aspects of plant development and growth, including in AR formation, to the best of our knowledge, there is a lack of information on this subject in olive. Therefore, this work aimed to analyze the expression pattern of several members of the *PIN* gene family in olive stem-base tissues during IBA-induced rooting of in vitro-cultured microshoots of cv. ‘Galega vulgar’, in order to contribute to a growing body of knowledge on the molecular mechanisms underlying this developmental process.

## 2. Results

### 2.1. Gene Expression Analysis

The expression of the putative *OePIN1a*-*c*, *OePIN2b*, *OePIN3a*-*c*, *OePIN5a*-*c*, *OePIN6*, and *OePIN8* transcripts was analyzed in both IBA-treated and nontreated microcuttings up to 17 days after inoculation.

**Figure 1:**
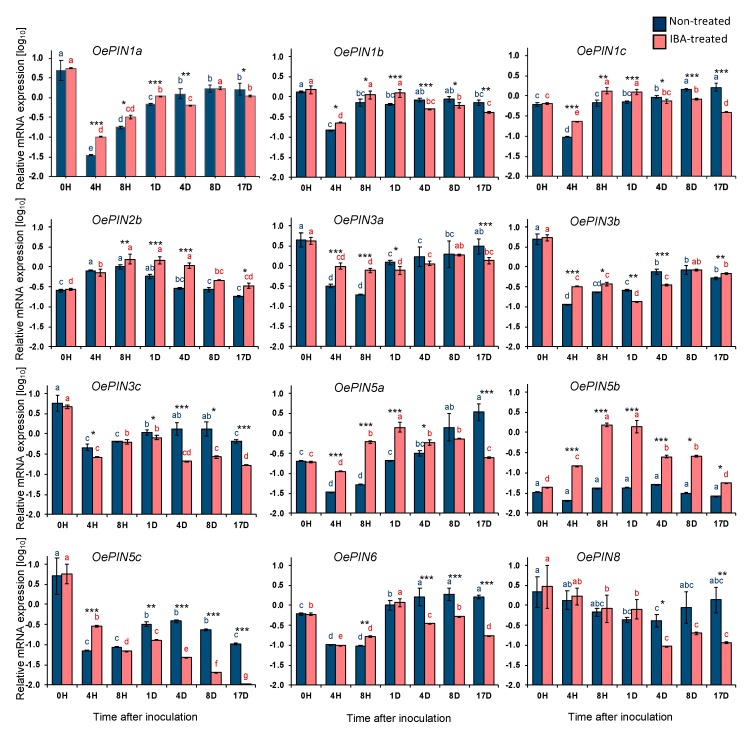
Relative mRNA expression of *OePIN* genes in stem basal segments of *Olea europaea* L. microcuttings during indole-3-butyric acid (IBA)-induced adventitious rooting. The relative expression values are depicted as the mean (Log10 transformed) ± the standard error of the mean (SEM) of four biological replicates (each consisting of 20 stem basal segments) for each time point. Distinct letters (*p* < 0.05) and asterisks (* *p* ≤ 0.05, ** *p* ≤ 0.01, *** *p* ≤ 0.001) indicate statistically significant differences. H: hours; D: days.

A downregulation in the expression of most of the transcripts was observed 4 h soon after inoculation in both nontreated (*OePIN1a:* 141-fold change, *p ≤* 0.001; *OePIN1b:* 9-fold change, *p ≤* 0.05; *OePIN1c:* 6.4-fold change, *p ≤* 0.001; *OePIN3a:* 14-fold change, *p ≤* 0.001; *OePIN3b*: 43-fold change, *p ≤* 0.05; *OePIN3c:* 12.5-fold change, *p ≤* 0.05; *OePIN5a*: 6-fold change, *p ≤* 0.05; *OePIN5c:* 72-fold change, *p ≤* 0.001; *OePIN6:* 5.8-fold change, *p ≤* 0.001) and IBA-treated (*OePIN1a:* 55-fold change, *p ≤* 0.05; *OePIN1b:* 6.6-fold change, *p ≤* 0.001; *OePIN1c:* 2.8-fold change, *p ≤* 0.001; *OePIN3a:* 4.3-fold change, *p ≤* 0.05; *OePIN3b*: 16.9-fold change, *p ≤* 0.05; *OePIN3c:* 17.5-fold change, *p ≤* 0.001; *OePIN5a*: 1.7-fold change, *p ≤* 0.001; *OePIN5c:* 20-fold change, *p ≤* 0.05: *OePIN6:* 6-fold change, *p ≤* 0.05) microcuttings (Figure 1). With the exception of *OePIN3c* and *OePIN6*, gene downregulation was less pronounced upon IBA treatment. Transcript downregulation was particularly drastic for *OePIN1a*, *OePIN3b*, and *OePIN5c*, within each of the corresponding gene subfamilies, with reductions over 40-fold changes in nontreated samples.

*OePIN1a*-*c* expression levels show significant differences between nontreated and IBA-treated microcuttings. Interestingly, and although the expression profile is similar between conditions, in IBA-treated samples, the expression of all three transcripts is higher than in nontreated samples from 4 h up to day 1 (i.e., during the induction phase). From day 4 until the end of the trial (i.e., during the initiation phase) this pattern was reversed and, overall, the expression of all three transcripts is lower in IBA-treated samples than in nontreated ones.

In contrast to most other genes analyzed here, *OePIN2b* exhibited a unique expression profile, being upregulated 4 h after inoculation with similar increments in both conditions, 3-fold change (*p ≤* 0.05) in nontreated explants, and 2.6-fold change (*p ≤* 0.001) in IBA-treated explants. From 8 h onwards, mRNA levels tended to decrease, reaching the basal expression levels in both conditions at the end of the rooting experiment.

Within the *OePIN5* gene subfamily, distinct expression profiles were observed for all three transcripts and between conditions within each transcript. *OePIN5b* showed the most interesting results, with the most relevant differences between the two conditions. While in nontreated samples *OePIN5b* mRNA levels remained stable throughout the rooting trial, in IBA-treated samples the expression of this gene was highly upregulated (35-fold change, *p ≤* 0.001) up to 8 h after inoculation, maintained up to day 1 and downregulated until the end of the rooting experiment when it reached the basal levels. On the other hand, the expression of *OePIN5a* in nontreated microcuttings raised from 4 h onwards, exceeding the basal expression levels, while in IBA-treated microcuttings an expression peak was observed around day 1, followed by a downregulation up to 17 days post inoculation, until basal levels were reached. Interestingly, both *OePIN5a* and *OePIN5b* had an expression peak around day 1 in IBA-treated samples but in nontreated samples the expression was similar to basal levels. Meanwhile, at 17 days after inoculation, *OePIN5b* reached basal expression levels in both conditions, while *OePIN5a* only reached these levels in the IBA-treated samples. On the contrary, *OePIN5c* mRNA remained below basal levels throughout the rooting experiment in both conditions. In contrast to nontreated samples, in IBA-treated samples this transcript decreased more sharply until the end of the trial, reaching levels tremendously low at 17 days post inoculation.

Regarding *OePIN6*, from 4 h up to day 1, mRNA levels were upregulated in both conditions. From day 1 onwards, the tendency was for *OePIN6* mRNA levels to decrease in IBA-treated samples, but to remain stable and above basal levels in nontreated samples until the end of the rooting induction experiment. This expression pattern was similar to that observed for *OePIN5a*.

Finally, and despite the high variability observed among samples in both conditions, there was a similar tendency for reduction of *OePIN8* transcript amounts in both conditions up to day 1. Subsequently, transcript amounts kept decreasing in IBA-treated samples, while in nontreated samples they recovered basal values until the end of the experiment.

### 2.2. ROS Accumulation in Stem-Base Tissues

**Figure 2:**
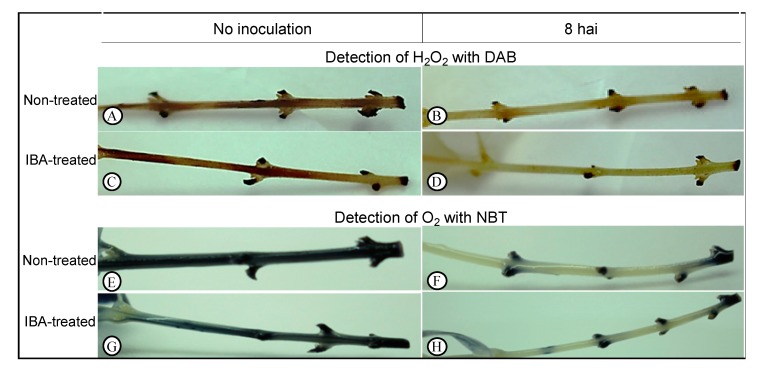
Detection of hydrogen peroxide (H_2_O_2_) with 3,3′-diaminobenzidine (DAB) (**A**–**D**) and superoxide anion (O_2_) with nitroblue tetrazolium (NBT) (**E**–**H**). Histochemical detection analyses were performed at 0 h after inoculation (hai) (no inoculation) and at 8 hai in the culture medium.

We evaluated the accumulation H_2_O_2_ and O_2_^•−^ in olive stem-base tissues using 3,3′-diaminobenzidine (DAB) and nitroblue tetrazolium (NBT) stainings, respectively, in nontreated and IBA-treated microcuttings (Figure 2). We found that soon after explant preparation for rooting (at 0 h, prior to inoculation) high staining intensity for both DAB (Figure 2A,C) and NBT (Figure 2E,G) substrates was observed in both types of stem, indicating high accumulation of ROS. Eight hours after inoculation, tissue discoloration was observed for both DAB (Figure 2B,D) and NBT (Figure 2F,H) chromogenic substrates, in both conditions, revealing that ROS levels decreased in nontreated and IBA-treated explants a few hours after inoculation

## 3. Discussion

Auxins play a central role in the control of adventitious rooting [38]. Trying to understand whether auxin transport could be impaired in difficult-to-root olive cultivars, we analyzed the expression of the olive *PIN*-formed gene family during IBA-induced in vitro adventitious root formation using the cultivar ‘Galega vulgar’.

To the best of our knowledge, this study shows for the first time the expression profile of the putative *OePIN1a*-*c*, *OePIN2b*, *OePIN3a*-*c*, *OePIN5a*-*c*, *OePIN6*, and *OePIN8* transcripts in olive stem-base tissues.

When looking at gene expression results in nontreated explants alone, our results indicate that most *OePIN* gene members were negatively affected by the explant preparation procedure, since gene expression was downregulated just a few hours after explant preparation and inoculation (Figure 1). This was observed for all transcripts analyzed, except for *OePIN2b*. This drastic downregulation might be related to the plant’s response to the stress situation (i.e., wounding) associated with explant preparation. In fact, the occurrence of high levels of ROS was demonstrated soon after explant preparation (prior to inoculation, at 0 hai) (Figure 2A,C,E,G), which are indicative of the presence of oxidative stress [57]. ROS are key signaling molecules that regulate growth and development and coordinate responses to biotic and abiotic stresses in plants [47]. The existence of cross-talk between ROS and auxin signaling has been reported [48], where stress-induced ROS production can alter auxin homeostasis [49,50] and also inhibit auxin-mediated signaling [55,58], while activating oxidative stress signaling [52]. ROS directly influence the action of auxins by a H_2_O_2_-dependent mitogen-activated protein kinase (MAPK) cascade that negatively affects auxin sensitivity by downregulating auxin-inducible gene expression [54]. On the other hand, auxins can induce the production of ROS [48] and regulate ROS homeostasis [51]. Therefore, ROS and auxins have been reported as regulators of plant development during stress [48,49,50]. ROS affect auxin signaling in different ways. Increased ROS levels may affect auxin signaling and homeostasis by affecting auxin biosynthesis, conjugation, degradation, and distribution [48,51,59]. Specifically, ROS can alter the directional transport of auxin through changes in the expression of genes encoding auxin transporters [60]. Indeed, it has been reported that ROS production under environmental stress can have an impact on auxin redistribution via repression of the polar auxin transporters [48]. An example is the data obtained by Blomster et al. [53] who demonstrated that apoplastic ROS negatively affect the expression of several transcripts encoding auxin efflux carriers. In line with these results, Pasternak and co-authors [51] showed that the expression of PIN1 and PIN3 genes decreases under oxidative stress-related growth conditions induced by alloxan. Another example involves sorghum (*Sorghum bicolor*), where the transcriptional analysis of auxin transporter genes revealed that their expression levels are affected by abiotic stresses, such as salt and drought [61], whereas cold stress impacts the intracellular trafficking of a subset of proteins, including PIN2 and PIN3, that inhibit auxin transport in the roots [62]. In our study, the correlation found between high ROS accumulation and downregulated *OePIN* genes corroborates those earlier studies reporting an impact of ROS on auxin transport. The crosstalk between ROS and auxins is on the basis of the so-called stress-induced morphogenic responses where stressed plants develop a general acclimation strategy, whereby plant growth is not ceased but instead redirected to diminish stress exposure. In fact, several events in developmental and growth processes are mediated by auxin signaling and the suppression of auxin signaling by mitochondrial ROS positions mitochondria as an important hub in the cellular signaling cascade that can block auxin signaling and thus play a central role in determining if cellular resources are used for growth (signaled by auxin) or stress resistance (signaled by ROS) [48,49,50]. With regard to AR formation, Santos Macedo and collaborators [63] have suggested that in olive this process is considered a stress-induced morphogenic response which involves reprogramming of the shoot cells [44,45,63].

When IBA was applied to the base of the olive stems, gene downregulation was less pronounced compared to nontreated stems, suggesting that auxin-sensitive cells are probably being induced to express *PIN* genes in response to the application of exogenous auxin. Nevertheless, this induction is not enough to overcome the stress impact on *PIN* gene downregulation observed in nontreated stems. Indeed, there are several studies reporting induced expression of *PIN* genes following exogenous auxin application. For instance, IBA induces *PIN1* during adventitious rooting in *Arabidopsis thaliana* thin cell layers [64]. Similarly, the genes *CpPIN1*, *CpPIN2*, *CpPIN3*, and *CpPIN4* increased their expression in response to IBA in *Carica papaya* [65]. In turn, maize genes *ZmPIN1a* and *ZmPIN1c* showed significantly upregulated expression after IAA treatment [66]. Likewise, *OsPIN1a* and *OsPIN1b* were induced by IAA in rice [67].

PIN1, PIN2, and PIN3 are located to the plasma membrane and play important roles in polar auxin transport [68]. In our study, we observed significant differences in *OePIN1a*-*c* expression levels between nontreated and IBA-treated microcuttings. While from 4 h up to day 1 (during the induction phase) the expression of all three transcripts is higher in IBA-treated samples, from day 4 until the end of the trial (during the initiation phase) the expression of all three transcripts is lower in IBA-treated samples, suggesting a reduction in auxin transport which probably has an effect on auxin redistribution throughout the stem-base tissues. This expression pattern was also observed for *OePIN3* (other *PIN* gene also playing important roles in PAT). Indeed, in nontreated microcuttings the expression profile of *OePIN3b* and *OePIN3c* was very similar throughout the rooting trial. However, in IBA-treated microcuttings the expression of *OePIN3b* and *OePIN3c* transcripts was quite different, particularly during the initiation phase (from 4 up to 14 days), suggesting that the application of exogenous auxins might be promoting a differential regulation of the direction in which PAT occurs. PIN1 has been associated with adventitious root development. For example, *OsPIN1* is involved in adventitious root emergence in rice [31] and *AtPIN1* is expressed during adventitious rooting in the hypocotyl of Arabidopsis seedlings and it induces auxin levels for AR initiation [69]. Additionally, Sukumar et al. [70] found that *PIN1* and *PIN3* mutations reduce AR formation in the hypocotyls of Arabidopsis seedlings.

*OePIN2b* showed an expression profile distinct from all other studied transcripts, being upregulated in both conditions instead of downregulated. It should be noted that this gene is the only one upregulated in nontreated stems, suggesting that it might be induced by wounding-related stress. In fact, PIN proteins are also involved in plant responses to stress situations [66,71,72], and PIN2 has even been referred to as a general stress target underlying the adaptive response of roots to abiotic stress [73]. Additionally, *OsPIN2* was found to be induced by cold stress and drought [74].

PIN5, a short PIN protein displaying a dramatically reduced central hydrophilic loop, is localized to endoplasmic reticulum (ER) where it mediates auxin flow from the cytoplasm into the ER lumen. AtPIN5 is pointed out to have a function in the regulation of intracellular auxin homeostasis and metabolism through subcellular compartmentalization [29,68,75]. Expression patterns differed considerably among the three *OePIN5* transcripts in nontreated explants, but when exogenous auxin was applied the expression profiles became very similar, particularly those of *OePIN5a* and *OePIN5b*, with a maximum expression around day 1, suggesting that the application of exogenous auxin may be regulating *OePIN5a* and *OePIN5b* transcripts in the same manner. The most interesting result involved *OePIN5b* whose expression increased considerably 8 h after inoculation in IBA-treated samples, suggesting the gene is likely being induced by exogenous auxin. Lu and collaborators [76], using rice transgenic lines with elevated and reduced *OsPIN5b,* also observed that *OsPIN5b* was induced by treatment with synthetic auxin. The same authors observed that transgenic lines overexpressing *OsPIN5b* presented high free IAA levels and less auxin conjugates, suggesting that OsPIN5b modulates auxin homeostasis leading to changes in auxin transport and distribution. In line with the results from Lu and collaborators [76], we also observed that upon IBA treatment the maximum expression peak for *OePIN5b* correlates with the maximum peak in free auxin levels observed by Porfirio et al. [34] using the same experimental system. In contrast, Mravec et al. [75] suggested that AtPIN5, by transporting auxin intracellularly from the cytoplasm into the ER lumen, reduces auxin availability for plasma-membrane-based auxin efflux, since they observed that PIN5 gain-of-function at the ER leads to lower free IAA levels. It is tempting to speculate that the application of exogenous auxin might regulate *OePIN5* transcripts, therefore affecting auxin homeostasis. This could lead to changes in auxin distribution and transport in the stem-base tissues, which may in turn play a role in the initiation of AR in olive microshoots. Additionally, the altered expression profile of *OePIN1* and *OePIN3* (both involved in PAT) in IBA-treated microshoots could also contribute to changes in auxin transport and distribution.

AtPIN5, AtPIN6, and AtPIN8 share the following features: a reduction of the central hydrophilic loop, ER location, and they all have been proposed to regulate auxin homeostasis [30,75,77,78,79]. Interestingly, in the present study, both *OePIN5a* and *OePIN6* showed a similar expression profile. In IBA-treated explants, both transcripts recover their basal expression levels before the end of the rooting trial (specifically, during the initiation phase), suggesting that the application of exogenous auxin might contribute to a retrieval of the basal expression levels observed at 0 hai, and therefore contribute to auxin homeostasis at this phase of the rooting process.

The present study also demonstrates that ROS levels were further reduced 8 h after inoculation in both nontreated and IBA-treated stems (Figure 2B,D,F,H), which might result from increased expression of the alternative oxidase (AOX) gene, as previously shown [80]. Consequently, this could lead to increased expression of the corresponding AOX protein, which has been reported to have a function in regulating ROS levels [81]. Given this function, AOX has been described to play a crucial role in redox regulation at a cellular level on environmental stresses [82]. Moreover, the production of ROS by mitochondria was suggested to be a critical factor for the induction of AOX [83,84,85]. Interestingly, Ivanova et al. [86] found a relationship between AOX and auxin efflux carriers. They showed that Arabidopsis *PIN1*, *PIN3*, *PIN4*, and *PIN7* genes were significantly downregulated when *AOX1a* was induced by antimycin A (an inhibitor of the mitochondrial electron transport chain). The authors suggested that *AOX1a* induction represses auxin cell-to-cell transport and auxin signaling. It is worth noting that our previous study [80], found a dramatic upregulation of the *OeAOX1a* gene in both nontreated and IBA-treated microcuttings. Taken together, our previous [80] and current findings show an association between ROS accumulation and a dramatic downregulation of most olive *PIN* genes, as well as induced expression of olive *AOX1* gene. This corroborates other previous studies [48,49,50,51,86] which describe an interplay between stress-induced ROS and auxin signaling.

In summary, we show here that the stress situation (caused by wounding) associated with explant preparation for rooting, and the resultant increased production of ROS, could impact on auxin transport and distribution through alterations in the expression of *OePIN* genes. Moreover, the application of exogenous auxin, through regulation of those genes, may play a role modulating auxin homeostasis and leading to auxin redistribution throughout the stem-base tissues, which may play an important role in AR formation. Further studies are required to elucidate the precise biological functions of the *PIN* gene family during AR formation in olive, namely, tissue-specific localization of these genes in olive stem-base tissues, as well as development of transgenic lines (overexpressing for example *OePIN5b*), which may provide new insights on the molecular mechanisms underlying this developmental process.

## 4. Materials and Methods

### 4.1. Plant Material and In Vitro Rooting Trials

*Olea europaea* L. plantlets of cv. ‘Galega vulgar’ (clone 1441) have been maintained under in vitro conditions since 2005 [87] and were used as the initial explant source for AR trials. Treatments for rooting induction were performed according to others [37] with some modifications. Briefly, stem cuttings (microcuttings) with four to five nodes were prepared from the upper part of in vitro-cultured plantlets and all leaves were removed except for the upper four. The base (approx. 1.0 cm) of each microcutting was immersed in a sterile solution of 14.7 mM IBA for 10 s [46]. Immediately after, microcuttings were aseptically inoculated in 500 mL glass flasks containing 75 mL semi-solid olive culture medium (OM), without plant growth regulators and supplemented with 7 g/L commercial agar-agar, 30 g/L d-mannitol, and 2 g/L activated charcoal [88]. Medium pH was adjusted to 5.8 prior to autoclaving (20 min at 121 °C, 1 kg/cm^2^). Twenty microcuttings were inoculated per flask and four flasks were used per time point. Time points used here corresponded to time after inoculation in the culture medium and corresponded to 0, 4, 8, 24 (1 day), 96 (4 days), 192 (8 days), and 408 (17 days) h. Time point 0 h refers to microcuttings that were immediately frozen and stored after preparation for rooting assay and, thus, were not inoculated in the culture medium. All cultures were kept in a plant growth chamber at 24 °C/21 °C (±1 °C) day/night temperatures, with a 15 h photoperiod, under cool-white fluorescent light at a photosynthetically active radiation (PAR) level of 36 µmol/m^2^ s^−2^ at culture height. During in vitro rooting experiments, the segments from the basal portion (approx. 1 cm from the base) of the microcuttings were collected from each flask at the time points indicated above. Basal segments from control microcuttings, prepared as described above, but not immersed in IBA, were also collected at the same time points and those corresponded to the nontreated microcuttings. All samples were flash frozen in liquid nitrogen and stored at −80 °C for subsequent analyses.

### 4.2. RNA Isolation and First-Strand cDNA Synthesis

Total RNA was isolated with the Maxwell 16 LEV simplyRNA purification kit (Promega, Madison, WI, USA) on a Maxwell 16 Instrument (Promega, Madison, WI, USA) according to the supplier’s instructions, and eluted in 50 µL of RNase-free water. The concentration of total RNA was determined with a NanoDrop-2000C spectrophotometer (Thermo Scientific, Wilmington, DE, USA), and RNA integrity was evaluated by agarose gel electrophoresis through visualization of the two ribosomal subunits in a Gene Flash Bio Imaging system (Syngene, Cambridge, UK). The GoScript Reverse Transcription System (Promega, Madison, WI, USA) was used to synthesize complementary DNA (cDNA) from RNA samples (using 1 µg of total RNA), according to manufacturer’s instructions.

### 4.3. Quantitative Real-Time PCR (qPCR)

Real-time PCR was performed with the Applied Biosystems 7500 Real-Time PCR System (Applied Biosystems, Foster City, CA, USA). Real-time PCR reactions were carried out using 1× Maxima SYBR Green qPCR Master Mix, 20 ng of cDNA, and specific primers (300 nM) for target and reference genes (Table 1), in a total volume of 18 µL. Specific primers were designed with Primer Express v3.0 (Applied Biosystems, Foster City, CA, USA) using default software settings, and were based on the putative Oe*PIN* members sequences obtained at the olive genome database (var. *europaea*, cv. Farga) (http://denovo.cnag.cat/genomes/olive/) using the Oe6 browser (Table 1). Phylogenetic analysis was performed using putative olive PIN sequences and 120 PIN proteins from 11 eudicot plant species (Hélia Cardoso, personal communication), according to others [89,90,91,92]. Actin (*OeACT*) and the elongation factor 1a (*OeEF1a*) were used as reference genes for expression data normalization. All primer pairs were checked for their probability to form dimers and secondary structures using the primer test tool of the software. The reactions were performed using the following thermal profile: 10 min at 95 °C, and 40 cycles of 15 s at 95 °C and 60 s at 60 °C. No-template controls (NTCs) were used to assess contaminations and primer dimers formation. A standard curve, with a total of four points, was performed using an undiluted pool containing all cDNA samples and three four-fold serial dilutions. All samples were analyzed in duplicate. Melting curve analysis was done to ensure amplification of the specific amplicon. Quantification cycle (Cq) values were acquired for each sample with the Applied Biosystems 7500 software (Applied Biosystems, Foster City, CA, USA).

### 4.4. Transcript Expression Analysis

For transcript expression levels normalization, Cq values were converted into relative quantities (RQ) by the delta-Ct method described by Vandesompele and co-authors [93]. The normalization factor was determined by the GeNorm algorithm [93], which corresponds to the geometric mean between the RQ of the selected reference genes for each sample. Finally, calculating the ratio between the RQ for each sample and the corresponding normalization factor, a normalized gene expression value was obtained for each gene of interest.

### 4.5. Histochemical Detection of Reactive Oxygen Species (ROS)

Twelve nontreated and IBA-treated microcuttings prior to inoculation (at 0 h) and 8 h after inoculation were used for ROS detection. Hydrogen peroxide (H_2_O_2_) was detected with 3,3′-diaminobenzidine (DAB) (Alfa Aesar, Karlsruhe, Germany), and superoxide anion (O_2_^•−^) was detected with nitroblue tetrazolium (NBT) (Alfa Aesar, Karlsruhe, Germany). The detection of H_2_O_2_ and O_2_^•−^ was performed as described by others [94,95,96] with some modifications. Briefly, for the detection of H_2_O_2_, microcuttings were immersed in 10 mM disodium hydrogen phosphate buffer pH 7.5 containing 0.05% (*v*/*v*) Tween 20 and 0.1% DAB; and for the detection of O_2_^•−^, microcuttings were immersed in 50 mM sodium phosphate buffer (prepared with sodium dihydrogen phosphate and disodium hydrogen phosphate) pH 7.5 containing 0.2% NBT. Microcuttings were incubated in DAB and NBT staining solutions in 50 mL falcon tubes (covered with aluminum foil) overnight at room temperature. Following incubation, the staining solutions were replaced by a chlorophyll removal solution containing ethanol, acetic acid, and glycerol in a proportion of (3:1:1, *v*/*v*) and microcuttings were then placed in a boiling water bath (at 95 °C) with intermittent shaking for 15 min to completely remove the chlorophyll from the tissues. The removal solution was replaced by a fresh one and microcuttings were allowed to stand for 30 min before photographs were taken. H_2_O_2_ was visualized as a dark brown precipitate and O_2_^•−^ as a dark blue precipitate in the tissue. Microcuttings immersed in 10 mM disodium phosphate buffer pH 7.5 and in 50 mM sodium phosphate buffer pH 7.5, without DAB and NBT, respectively, were used as controls.

### 4.6. Statistical Analysis

The graphs of relative mRNA expression levels show the mean (log_10_ transformed) ± standard error of the mean (SEM) of four biological replicates (each consisting of a pool of 20 stem basal segments) for each time point and for each experimental condition (nontreated and IBA-treated). Statistical significances among different time points after inoculation were evaluated by one-way analysis of variance (ANOVA, *p* < 0.05), followed by Tukey’s honest significant difference (HSD) post-hoc test (*p* < 0.05), when normality (by Shapiro–Wilk test) and/or homogeneity of variances (by Bartlett’s test) were verified. When the assumptions of one-way ANOVA test were not met, the Kruskal–Wallis test (*p* < 0.05) followed by the Pairwise Wilcoxon Rank Sum test (also called Mann–Whitney test) (*p* < 0.05) were used instead. Student’s *t*-test (*p ≤ 0.05, **p ≤ 0.01, ***p ≤ 0.001) was adopted when comparing between conditions (no treatment vs. treatment with IBA) within the same time point and when normality (by Shapiro–Wilk test) and/or homogeneity of variances (by Bartlett’s test) were verified. Otherwise, the unpaired two-samples Wilcoxon test (*p ≤ 0.05, **p ≤ 0.01, ***p ≤ 0.001) was applied. Distinct letters indicate statistically significant differences (*p* < 0.05) within the same condition. All statistical analyses were performed using the computing environment R [97].

## Figures and Tables

**Table 1:** Primers used for RT-qPCR analysis designed based on *PIN* sequences retrieved from *Olea euroapaea* subsp. *europaea* var. *europaea* (cv. ‘Farga’).

Gene	Locus ID	Primer Sequences 5′→3′	Amplicon Size (bp)
*OePIN1a*	OE6A110180	Fw: TATGAGAAGGGCAGTAGAAGATTAGAGCAT Rv: GGGCCAATGAATTCCGTTTC	86
*OePIN1b*	OE6A100299	Fw: TGCTGGGATTATGAAAAGGAA Rv: TTGGTGATCCCAACTTCAAA	82
*OePIN1c*	OE6A008174	Fw: GGCTATGAATGTGATGTGTCGAT Rv: TTTCGGTGTCCAAGTCTTTGA	64
*OePIN2b*	OE6A029229	Fw: CTTCTTGGGGTGTAACTTTGG Rv: AAAAACAAGCAACAAGAACATCA	147
*OePIN3a*	OE6A013411	Fw: TTTGGAATGTTGATTGCATTG Rv: TTCAACGAAGCGTCACAATC	128
*OePIN3b*	OE6A121027	Fw: TCCCGATTACGCTCGTCTAT Rv: TTTGACACCATTTTCACACAGTC	84
*OePIN3c*	OE6A040519	Fw: ATCGCGCTACCGATTACACT Rv: CCCGAATATGACCAAGAACAA	72
*OePIN5a*	OE6A089248	Fw: CGTCATTTTAGATAGTATCCATTGATGT Rv: TTTATACTGGAAAGTCCTAGTCAGCA	53
*OePIN5b*	OE6A062743	Fw: GCTTCCACTCTTGATTGGCTA Rv: TGGATCGTCAGATGCAAACT	82
*OePIN5c*	OE6A015595	Fw: GGTGTTGATCGGATATTATGCAGTT Rv: ATGATCCAAAATTTAGCCATGAAAAC	125
*OePIN6*	OE6A074435	Fw: CCCGTGACCCTTGTCTATTACATAT Rv: TTATCCTTTTCCTTTTTGTTCCAACT	72
*OePIN8*	OE6A113148	Fw: GCCAATTGCATTAGCCTACTACTTC Rv: GCACGAGATGAATCTTGTGTTATCA	74

Locus ID retrieved from http://denovo.cnag.cat/genomes/olive/ (Oe6 browser).
